# Supplementary material for: Care to share? Experimental evidence on code sharing behavior in the social sciences
Source: PLoS One. 2023 Aug 7;18(8):e0289380. doi: 10.1371/journal.pone.0289380 (PMC10406284; doi:10.1371/journal.pone.0289380)
Supplement: S1 File — (PDF) [file pone.0289380.s001.pdf]

Care to Share? Determinants of Code Sharing Behavior in the Social Sciences

— Supporting Information —

Daniel Krähmer, Laura Schächtele, Andreas Schneck

Tables

Table A Literature overview . . . 2
Table B Deviations from preregistration . . . 3
Table C z-tests of proportions (main results) . . . 3
Table D Linear probability model with two-way interactions . . . 4
Table E z-tests of proportions (robustness checks) . . . 4

Figures

Figure A Documentation of data collection . . . 6
Figure B Exemplary email wording . . . 7
Figure C Response rates across treatment conditions . . . 8

Other

Text A Statistical power considerations . . . 8

Table A. Literature overview. Previous Literature on Code and Data Sharing in Academia

Study	Discipline	Target Sample	Requestor	Sample Size	Request for		Sharing Rate	Request Wording
					Data	Code		
[1]	Informatics	articles from the Association for Computing Machinery	individual researcher(s)	263		✓	33.1%	email text available via tecnical report
[2]	Psychology	articles from four psychology journals	individual researcher(s)	53	✓		37.7%	
[3]	Economics	articles from the Journal of Money, Credit and Banking (1980 or later)	research group	154	✓	✓	34.4%	
[4]	Cancer biology	high-impact articles (2010-2012) *	research group	53 studies 193 experiments	✓		18.1%	
[5]	Psychology, Psychiatry	high-impact articles from psychology and psychiatry (2006-2011)	individual researcher(s)	111	✓		13.5%	initial request available via OSF
[6]	Economics	articles from economics and business (2009), restriction on data availability statement	individual researcher(s)	200	✓	✓	44.0%	email text included in the article
[7]	Cell Cancer	articles from MEDLINE and EMBASE *	individual researcher(s)	79	✓		53.2%	
[8]	Genetics	articles from four psychology journals (2012)	individual researcher(s)	30	✓		3.3%	
[9]	Economics	articles from the American Economic Review (1997-2002) *	individual researcher(s)	8	✓	✓	50.0%	
[10]	Marketing	articles from five marketing journals (1978-1979)	individual researcher(s)	99	✓		49.5%	initial request included in the article
[11]	Medicine	articles from PLoS journals	individual researcher(s)	10	✓		10.0%	
[12]	Political Science	articles from four political science journals (2015)	individual researcher(s)	132	✓	✓	52.3%	
[13]	Multidisciplinary	articles from Science magazine (2011 - 2012)	individual researcher(s)	180	✓	✓	36.1%	initial request and reminder available via GitHub
[14]	Multidisciplinary	articles from Nature and Science (2000-2009, 2010-2019)	research group	310	✓		39.4%	initial request available via publisher
[15]	Psychology	articles from three psychology journals (2012)	research group	394	✓		37.6%	initial request available via OSF
[16]	Genetics	Web of Science articles (2011-2012) *	research group	57	✓		59.6%	
[17]	Biology	journal articles (1992 - 2012) *	research group	516	✓		19.6%	initial request and reminder available via publisher
[18]	Psychology	articles from four APA journals (2004)	individual researcher(s)	141 articles 249 datasets	✓		25.7%	
[19]	Psychology	articles from APA journals (1959-1961)	student	37	✓		24.3%	

* Additional restriction on a topic or method.

Table B. Deviations from preregistration. Unless specified below, all research steps have been carried out according to the preregistered protocol available via OSF (https://osf.io/bqjcz).

	Preregistered	Realized	Justification/Description
Coding of outcome	binary	binary/categorial	In line with the preregistration, the central outcome variable was dummy-coded for all main analyses. To allow for more in-depth description, we additionally created a categorical outcome variable that captured the timing of authors' responses (after initial request, after $1^{st}/2^{nd}/3^{rd}$ reminder).
Debriefing	via email	via email/homepage	To improve accessibility, we shortened our debriefing email and outsourced further information to our project homepage. In the email, we explicitly debriefed authors on the experimental nature of our request and pointed them towards the homepage.
Language	English	English/German	If authors replied to our code request in German, we decided to switch language for all further personalized correspondence. Any outstanding general emails (i.e. reminders), however, were written in English in accordance with the preregistration.
Sample size	1207	1028	We deleted an empty row (n=1) in the ESS database which had gone unnoticed at the time of preregistration. As prespecified, we treated researchers whom we ultimately could not reach as sample-neutral failure (n=95). We also excluded researchers that appeared in the ESS database without having used ESS data substantially (i.e. overcoverage, n=83).

Table C. z-tests of proportions (main results). None of our treatments yields a statistically significant effect in the expected direction.

	Treatment		Control		Test of proportions		
	N	Proportion	N	Proportion	Δ	z	p
Framing[a]	520	0.340	508	0.409	-0.069	2.287	0.989
FAIR[b]	261	0.395	261	0.383	0.011	-0.269	0.394
Altruism[b]	255	0.333	261	0.383	-0.050	1.180	0.881
Citation[b]	251	0.386	261	0.383	0.003	-0.077	0.469
Effort[c]	514	0.375	514	0.374	0.002	-0.064	0.474

[a] We consider positive framing as treatment, and negative framing as control.
[b] For all appeal treatments, we consider the neutral baseline request as control.
[c] We consider the neutral baseline request without any information on code cleaning requirements as control.

Table D. Linear probability model with two-way interactions. All coefficients from the interacted LPM are statistically insignificant (dependent variable: Code shared 0/1).

	Coefficient	S.E.
**Framing (Ref.: Negative)**		
Positive	−0.013	(0.067)
**Appeal (Ref.: No information)**		
FAIR	0.094	(0.074)
Altruism	−0.031	(0.074)
Citation	0.008	(0.075)
**Effort (Ref.: No information)**		
No Effort	0.028	(0.067)
**Interaction Terms**		
Positive X FAIR	−0.107	(0.085)
Positive X Altruism	0.001	(0.085)
Positive X Citation	−0.058	(0.086)
Positive X No Effort	−0.030	(0.060)
No Effort X FAIR	−0.058	(0.085)
No Effort X Altruism	−0.038	(0.085)
No Effort X Citation	0.049	(0.086)
Constant	0.383***	(0.054)
Observations	1028	

* $p < 0.05$, ** $p < 0.01$, *** $p < 0.001$

Table E. z-tests of proportions (robustness checks). All treatment effects remain stable across reasonable sample restrictions.

Effect of framing treatment (ref. negative)

	Treatment		Control		Test of proportions		
	N	Proportion	N	Proportion	Δ	z	p
Main results (full sample)	520	0.340	508	0.409	-0.069	2.287	0.989
Robustness: Exclusion of...							
...authors with queries	466	0.322	455	0.393	-0.072	2.265	0.988
...double requests	498	0.337	481	0.397	-0.060	1.939	0.974
...custom requests	516	0.341	497	0.412	-0.071	2.345	0.990
...previously shared	503	0.326	492	0.396	-0.070	2.309	0.990
...all unusual cases	432	0.303	419	0.368	-0.064	1.987	0.977

Effect of FAIR treatment (ref. no specific appeal)

	Treatment		Control		Test of proportions		
	N	Proportion	N	Proportion	Δ	z	p
Main results (full sample)	261	0.395	261	0.383	0.011	-0.269	0.394
Robustness: Exclusion of...							
...authors with queries	237	0.380	236	0.356	0.024	-0.537	0.296
...double requests	250	0.396	248	0.375	0.021	-0.481	0.315
...custom requests	255	0.396	257	0.381	0.015	-0.342	0.366
...previously shared	251	0.375	250	0.360	0.015	-0.337	0.368
...all unusual cases	218	0.362	213	0.319	0.043	-0.944	0.172

Effect of altruism treatment (ref. no specific appeal)

	Treatment		Control		Test of proportions		
	N	Proportion	N	Proportion	Δ	z	p
Main results (full sample)	255	0.333	261	0.383	-0.050	1.180	0.881
Robustness: Exclusion of...							
...authors with queries	227	0.313	236	0.356	-0.043	0.984	0.837
...double requests	240	0.321	248	0.375	-0.054	1.256	0.895
...custom requests	253	0.336	257	0.381	-0.045	1.068	0.857
...previously shared	251	0.327	250	0.360	-0.033	0.785	0.784
...all unusual cases	212	0.297	213	0.319	-0.022	0.493	0.689

Effect of citation treatment (ref. no specific appeal)

	Treatment		Control		Test of proportions		
	N	Proportion	N	Proportion	Δ	z	p
Main results (full sample)	251	0.386	261	0.383	0.003	-0.077	0.469
Robustness: Exclusion of...							
...authors with queries	221	0.380	236	0.356	0.024	-0.535	0.296
...double requests	241	0.373	248	0.375	-0.002	0.036	0.514
...custom requests	248	0.391	257	0.381	0.010	-0.226	0.410
...previously shared	243	0.383	250	0.360	0.023	-0.522	0.301
...all unusual cases	208	0.361	213	0.319	0.041	-0.895	0.185

Effect of effort treatment (ref. no information on code cleaning requirements)

	Treatment		Control		Test of proportions		
	N	Proportion	N	Proportion	Δ	z	p
Main results (full sample)	514	0.375	514	0.374	0.002	-0.064	0.474
Robustness: Exclusion of...							
...authors with queries	458	0.365	463	0.350	0.015	-0.467	0.320
...double requests	487	0.363	492	0.370	-0.006	0.210	0.583
...custom requests	506	0.379	507	0.373	0.007	-0.219	0.413
...previously shared	497	0.358	498	0.363	-0.005	0.174	0.569
...all unusual cases	419	0.332	432	0.338	-0.006	0.192	0.576

Figure A. Documentation of data collection. The figure provides daily information on the inflow and outflow of emails from 6 July 2022 to 17 January 2023.

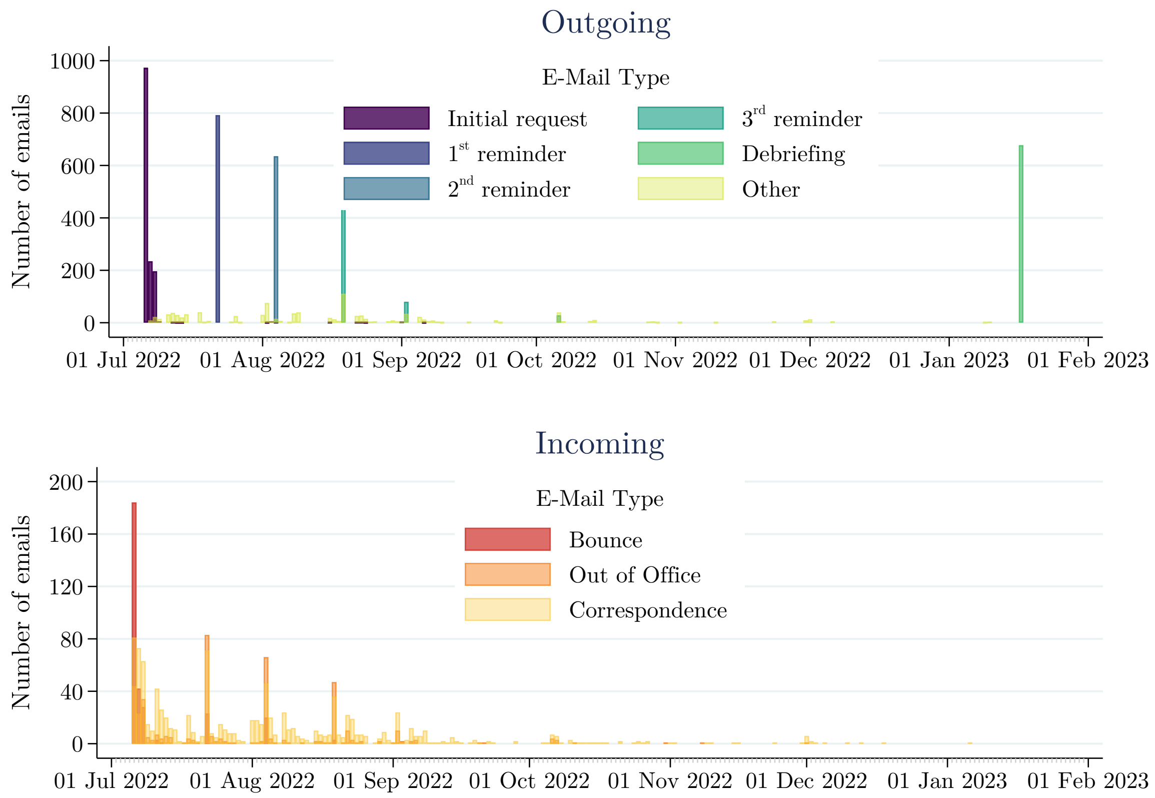

Figure B. Exemplary email wording. Treatments are underlined, brackets indicate personalized text modules. All email drafts can be accessed online (https://osf.io/bqjcz).

Dear [first name initial] [last name],

I hope this e-mail finds you well, despite the difficult times we are currently facing.

My name is Daniel Krähmer and I am a doctoral researcher at the chair for Quantitative Social Research under Prof. Dr. Katrin Auspurg at the LMU Munich. I am reaching out to you as part of the Priority Program META-REP funded by the German Research Foundation (DFG), **to kindly request your research code for your [publication year] article "[title]", published in [publication outlet]**.

To further enhance the quality, relevance, and success of social science research, our project aims to assess the reproducibility of randomly selected articles from the European Social Survey's bibliographic database. Would you mind sharing your code with us to make sure your article can be included in our analysis? In case we have overlooked available replication files for your article online, or if you are not the right person to contact, please point us in the right direction.

Please note:

- By providing access to your code, you honor the FAIR Guiding Principles (Wilkinson et al. 2016) and showcase your commitment to good scientific practice. The FAIR principles aim to make research more transparent and sustainable, and have been adopted by research institutions worldwide.
- Ideally, you would provide access to the entire code, starting from the publicly available ESS files. This includes everything from initial data preparation to final results. Do not worry about any further preparation or code cleaning, this is not required.
- Should your analysis feature data other than the ESS, please provide access to these data along with your research code, if possible. This will greatly simplify any replication efforts.
- Our primary focus is on aggregated replication rates of published results; we will therefore not disclose your individual code without your permission. Should our results substantially differ from yours, we would be happy if we may get back to you.

Your cooperation is greatly appreciated and will substantially benefit our research.

We have compiled some background information below. Please do not hesitate to contact us with any questions or concerns.

Kind regards on behalf of the entire team,
Daniel Krähmer

- - -

Daniel Krähmer, M.A. | LMU Munich | Department of Sociology | Chair for Quantitative Social Research (Prof. Dr. Katrin Auspurg) | Konradstr. 6 | DE-80801 Germany

Background Information:
The project "Enhancing the Robustness of Observational Social Science Research by Computational Multi-Model Analyses" is part of the DFG Priority Program META-REP. One of the project's main goals is to quantify both the reproducibility of observational social science research and various reasons underlying (non)reproducibility. While there is currently much debate surrounding replications, it tends to go unnoticed that studies may not be replicable for a number of reasons. This includes honest errors, different sample or model specifications, different operationalizations, as well as software changes outside of the individual researcher's control. Often, reproductions fail from the outset due to missing data and code, although journals now frequently require researchers to share this material at publication.

This project focuses on articles using data from the European Social Survey (ESS). As the ESS constitutes a major public resource that you have benefitted from in the past, we hope you will be sympathetic to our request. We are convinced that this project will provide valuable insights on the replicability of social science research and will benefit both the research community and the general public.

Figure C. Response rates across treatment conditions. The figure plots mean response rates for all 16 distinct treatment conditions in ascending order. Black/gray squares represent active/passive treatment conditions, respectively.

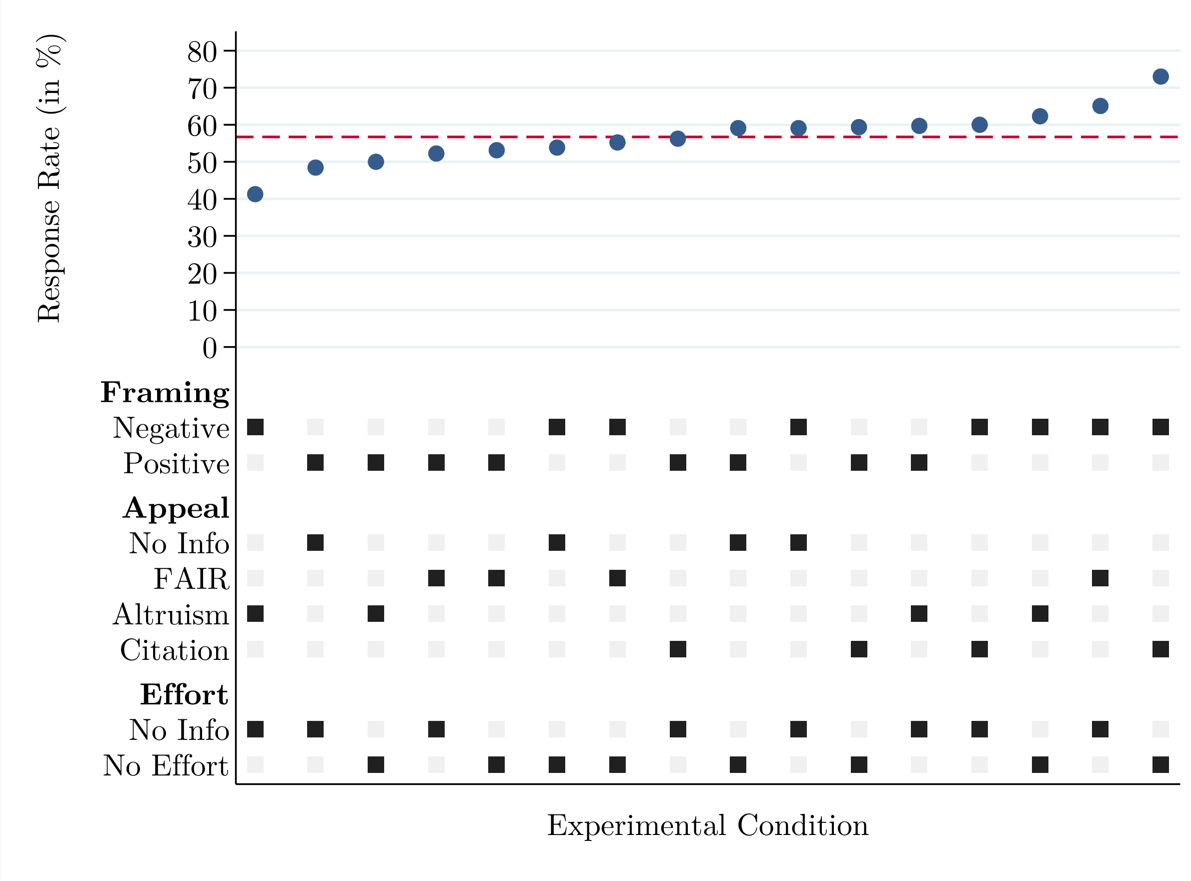

Text A. Statistical power considerations. Given our sample size and the observed baseline sharing rate of about 40%, the experiment's statistical power would be rather poor if one assumes the effect to be very small, i.e. our intervention leading to a 5 percentage points increase in code sharing rates (Cohen's d = 0.1). In that case, the statistical power for the effort and framing treatment would be 49.1% (N = 1,028) and only 30.9% for the appeal treatment (N = 514). Assuming a small effect, i.e. our intervention leading to an increase in code sharing rates by 10 percentage points ($d = 0.2$), statistical power is already quite high (94.3% for the binary treatments, 73.8% for the four-level treatment). Finally, for upper medium ($d = 0.4$) and large effect sizes ($d = 0.8$), the statistical power is excellent for all treatments (>99%). Admittedly, these power estimates rely on the assumption of no interaction effects between treatments, i.e. that we tested our main effects in different (independent) samples. If this condition is not met, statistical power would be lower than estimated. However, neither theoretical considerations nor results from the interacted linear probability model reported in Tab. D point towards interactions playing any meaningful role in our study. Taken together, our field experiment's statistical power seems more than adequate (i.e. >80%, [20]) to detect any main effect that would improve code sharing behavior substantially.

References

[1] Collberg C, Proebsting TA. Repeatability in Computer Systems Research. Communications of the ACM. 2016;59(3):62–69. doi:10.1145/2812803.

[2] Craig JR, Reese SC. Retention of Raw Data: A Problem Revisited. American Psychologist. 1973;28(8):723. doi:10.1037/h0035667.

[3] Dewald WG, Thursby JG, Anderson RG. Replication in Empirical Economics: The Journal of Money, Credit and Banking Project. The American Economic Review. 1986;76(4):587–603.

[4] Errington TM, Mathur M, Soderberg CK, Denis A, Perfito N, Iorns E, et al. Investigating the Replicability of Preclinical Cancer Biology. eLife. 2021;10:e71601. doi:10.7554/eLife.71601.

[5] Hardwicke TE, Ioannidis JPA. Populating the Data Ark: An Attempt to Retrieve, Preserve, and Liberate Data from the Most Highly-Cited Psychology and Psychiatry Articles. PLOS ONE. 2018;13(8):e0201856. doi:10.1371/journal.pone.0201856.

[6] Krawczyk M, Reuben E. (Un)Available upon Request: Field Experiment on Researchers' Willingness to Share Supplementary Materials. Accountability in Research. 2012;19(3):175–186. doi:10.1080/08989621.2012.678688.

[7] Kyzas PA, Loizou KT, Ioannidis JPA. Selective Reporting Biases in Cancer Prognostic Factor Studies. JNCI: Journal of the National Cancer Institute. 2005;97(14):1043–1055. doi:10.1093/jnci/dji184.

[8] Leberg PL, Neigel JE. Enhancing The Retrievability Of Population Genetic Survey Data? An Assessment Of Animal Mitochondrial DNA Studies. Evolution. 1999;53(6):1961–1965. doi:10.1111/j.1558-5646.1999.tb04576.x.

[9] McCullough BD, Vinod HD. Verifying the Solution from a Nonlinear Solver: A Case Study. American Economic Review. 2003;93(3):873–892. doi:10.1257/000282803322157133.

[10] Reid LN, Rotfeld HJ, Wimmer RD. How Researchers Respond to Replication Requests. Journal of Consumer Research. 1982;9(2):216–218. doi:10.1086/208916.

[11] Savage CJ, Vickers AJ. Empirical Study of Data Sharing by Authors Publishing in PLoS Journals. PLOS ONE. 2009;4(9):e7078. doi:10.1371/journal.pone.0007078.

[12] Stockemer D, Koehler S, Lentz T. Data Access, Transparency, and Replication: New Insights from the Political Behavior Literature. PS: Political Science & Politics. 2018;51(4):799–803. doi:10.1017/S1049096518000926.

[13] Stodden V, Seiler J, Ma Z. An Empirical Analysis of Journal Policy Effectiveness for Computational Reproducibility. Proceedings of the National Academy of Sciences. 2018;115(11):2584–2589. doi:10.1073/pnas.1708290115.

[14] Tedersoo L, Küngas R, Oras E, Köster K, Eenmaa H, Leijen Ä, et al. Data Sharing Practices and Data Availability upon Request Differ across Scientific Disciplines. Scientific Data. 2021;8(1):192. doi:10.1038/s41597-021-00981-0.

[15] Vanpaemel W, Vermorgen M, Deriemaecker L, Storms G. Are We Wasting a Good Crisis? The Availability of Psychological Research Data after the Storm. Collabra. 2015;1(1):3. doi:10.1525/collabra.13.

[16] Vines TH, Andrew RL, Bock DG, Franklin MT, Gilbert KJ, Kane NC, et al. Mandated Data Archiving Greatly Improves Access to Research Data. The FASEB Journal. 2013;27(4):1304–1308. doi:10.1096/fj.12-218164.

[17] Vines TH, Albert AYK, Andrew RL, Débarre F, Bock DG, Franklin MT, et al. The Availability of Research Data Declines Rapidly with Article Age. Current Biology. 2014;24(1):94–97. doi:10.1016/j.cub.2013.11.014.

[18] Wicherts JM, Borsboom D, Kats J, Molenaar D. The Poor Availability of Psychological Research Data for Reanalysis. American Psychologist. 2006;61(7):726–728. doi:10.1037/0003-066X.61.7.726.

[19] Wolins L. Responsibility for Raw Data. American Psychologist. 1962;17(9):657–658. doi:10.1037/h0038819.

[20] Cohen J. Statistical Power Analysis for the Behavioral Sciences. Second edition ed. Hillsdale, N.J: L. Erlbaum Associates; 1988.
