# Supplementary material for: Care to share? Experimental evidence on code sharing behavior in the social sciences
Source: PLoS One. 2023 Aug 7;18(8):e0289380. doi: 10.1371/journal.pone.0289380 (PMC10406284; doi:10.1371/journal.pone.0289380)
Supplement: Attachment — Submitted filename: Rebuttal.pdf [file pone.0289380.s002.pdf]

LMU LUDWIG-MAXIMILIANS-UNIVERSITÄT MÜNCHEN

Daniel Krähmer, M.A.

Telephone +49 (0) 89 2180-2929

daniel.kraehmer@soziologie.uni-muenchen.de

Address
Chair Prof. Dr. Katrin Auspurg
Konradstraße 6, Raum 412
80801 München
Germany

Munich, 30 June 2023

Dear Editors,

We thank you and the two anonymous reviewers for their helpful comments that substantially improved our manuscript. We have edited our submission to address all suggestions and concerns that were raised during the review process.

Below is a detailed point-by-point response.

We hope the revised manuscript is now suitable for publication in PLOS One and are very much looking forward to your decision.

Thank you and best wishes also on behalf of my co-authors,

Daniel Krähmer

Reply to the Editors:

1. Please ensure that your manuscript meets PLOS ONE's style requirements, including those for file naming.

   We modified our manuscript's title page and rearranged the sections "Acknowledgments", "References", and "Supporting Information" to meet PLOS One's style requirements. All files are named correctly and the manuscript was prepared using PLOS One's LaTeX template.

2. We note that you have stated that you will provide repository information for your data at acceptance. Should your manuscript be accepted for publication, we will hold it until you provide the relevant accession numbers or DOIs necessary to access your data. If you wish to make changes to your Data Availability statement, please describe these changes in your cover letter and we will update your Data Availability statement to reflect the information you provide.

   Our data is currently stored on a private OSF repository and has been made available to the reviewers through an access link provided in the manuscript. Upon acceptance, the OSF repository will be made public and we will be happy to provide PLOS One with the relevant DOI.

3. We note that you have provided funding information that is not currently declared in your Funding Statement. However, funding information should not appear in the Acknowledgments section or other areas of your manuscript. We will only publish funding information present in the Funding Statement section of the online submission form.

   In line with your recommendations, we have revised both the Acknowledgments section and our Funding Statement:

   > "Acknowledgements: We thank Katrin Auspurg, who has been intimately involved in planning and conducting the experiment. Thanks to two anonymous reviewers for their helpful comments and to Richard Vielberg for superb research assistance. Lastly, we are indebted to all researchers who participated in our experiment and went to great lengths to share their research code with us."

   > "Funding Statement: This research has been funded by the German Research Foundation (www.dfg.de/en) through the Priority Program META-REP (Project 464507200). The funders had no role in study design, data collection and analysis, decision to publish, or preparation of the manuscript."

Reply to Reviewer #1:

*** Major Comments ***

1. Please include a discussion of the duplicitous researcher. If I am a duplicitous researcher, and I receive an email (as described) stating the following: "our project aims to assess the reproducibility of randomly selected articles from the European Social Survey's bibliographic database. Would you mind sharing your code with us to make sure your article can be included in our analysis?" I will simply ignore it. What implications does this have for the paper's results? To my reading, this will mean that those who reply are more likely to be honest researchers. The estimated nudge effects are then lower bounds, should the proportion of duplicitous researchers shrink in the future (who would not be nudge-able ever).

   In line with your suggestion, we included a discussion on the role of duplicitous researchers. The respective section in the manuscript now reads:

   > "…simple nudges are clearly not sufficient to influence researchers' code sharing behavior. Our estimates might represent a somewhat lower bound of the true nudging effect, though, as some participants could have been impervious to nudging *per se*. This applies, for instance, to researchers engaging in questionable research practices who would have ignored our request regardless of its wording. We expect the number of duplicitious researchers in our sample to be low but have no way of testing this assumption." (l. 368-374).

2. While Table D of the supplementary information is almost comprehensive, I would like to see the following: Line 234 mentions a strong assumption. " Researchers who promised to share their code but failed until our deadline were coded as unwilling to share." Are the results robust to their exclusion? Are results robust to their coding as 1? This proportion of researchers seemed non-trivial.

   Although this was not the main point, your helpful comment prompted us to streamline the wording throughout the manuscript to reflect that our field experiment captured actual sharing *behavior* rather than abstract sharing *intentions* ("willingness to share"). The line in question therefore now reads:

   > "Researchers who promised to share their code but failed until our deadline were coded as non-compliant (n = 28)." (l. 243-244).

   Because the experiment targets researchers' actual *behavior*, we believe that coding non-compliant authors as 1 solely based on their stated intentions would blur our estimand and

reduce conceptual clarity. This is even more true as none of the authors who had put us off ended up sharing their code with us, not even months after the deadline.

From a purely practical standpoint, excluding these cases should leave results unchanged due to the small number of cases (n = 28). To increase readers' confidence in our analytical strategy, the revised manuscripts includes the respective case number (see above).

3. Please answer: By treatment, is response rate different? I understand that the final dummy variable is 0 when not and 1 when the code was shared, but response rate could tell the reader something more. The data is already well anonymized so I do not see this being an issue of privacy for this manuscripts authors.

In line with your suggestion, we added Figure C to the supplement, which plots the mean response rate across experimental conditions. Naturally, response rates are consistently higher than code sharing rates (min: 41.3%, max: 73,0%, mean: 57.6%). As we did not formulate any hypotheses regarding our treatments' impact on *response* behaviour, we opted for a purely descriptive depiction and refrained from statistical significance testing. The revised manuscript now includes the following reference:

> "As Fig. 6 shows, code sharing rates ranged from about 20% to 50% across unique treatment conditions (for a depiction of *response* rates across treatment conditions, see Figure C in S1 File)." (l. 344-346)

4. What is the proportion of authors that needed clarification about what research code is? Line 268. Is this extra coaching applied evenly between treatments?

The revised manuscript includes the number of researchers who needed clarification what was meant by research code:

> "If authors indicated confusion about what we meant by research code (n = 22), we sent a follow-up email providing examples and clarification." (l. 279-281)

The number of authors needing clarification varied from 0 to 5 across treatment conditions and did not point to any meaningful regularity, i.e. certain text modules being more/less clear.

5. There is an assumption being made here about no interactivity between nudges. I would like to see a simple regression examining this, using interaction effects. A simple linear probability model would suffice in my opinion.

The revised supplement includes a linear probability model with all two-way interactions. We point readers to this table in the note of Figure 6, which now reads:

> "Code sharing rates for all 16 distinct treatment conditions with 95% confidence intervals. The treatment conditions are sorted in ascending order. Black/gray squares represent active/passive treatment conditions, respectively. Results from a linear probability model with all two-way interactions are reported in Tab. D in S1 File."

6. The post-hoc achieved power for the framing treatment is 62.7%. However, this includes non-response. From G*Power we have...

   z tests - Proportions: Difference between two independent proportions

   Analysis: Post hoc: Compute achieved power

   Input: Tail(s) = Two

   Proportion p2 = 0.409

   Proportion p1 = 0.340

   α err prob = 0.05

   Sample size group 1 = 508

   Sample size group 2 = 520

   Output: Critical z = 1.9599640

   Power (1-β err prob) = 0.6277340
   For the framing statistical test. I would like to see calculations of post-hoc achieved power for the remainder of the hypotheses.

   We appreciate the remark and added a section on statistical power to the manuscript. More specifically, we engaged in power calculations based on plausible effect sizes instead of running post-hoc power analyses. Our reasoning is the following:

   The statistical power is a conditional probability of finding a significant effect given the existence of an effect (H_1 being true). In post-hoc power analysis, it is impossible to distinguish if the observed effect is, indeed, a real existing effect or just random variation around H_0. Any post-hoc power calculation would need to rely on the assumption that the true effect is identical to the one observed. In our field experiment, effects were mostly negligible which, as we argue in the article, points to no underlying true effect (H_0 being true). In the most extreme case, taking these null effects as the starting point for a post-hoc power analysis would yield a statistical power of 0.05 (identical to α = 5%) because the conditional probability of finding a significant effect in case there is no underlying true effect is equivalent to alpha. Ultimately, post-hoc power is a direct function of the

p-value and does not provide any additional information as such (see Lakens 2022 [10.1525/collabra.33267]).

Having said this, we do see the point of commenting on the issue of statistical power in the manuscript and added the following sentence:

> "Assuming a moderately large effect, i.e. our treatment leading to an increase in code sharing rates by 15 percentage points (*d* = 0.3), the statistical power for both two-level and four-level treatments is more than adequate (96.25% and 99.93%, respectively; see Text A in S1 File for a detailed discussion on statistical power)." (l. 252-256)

The respective section in the Supplemental Material reads:

> "Given our sample size and the observed baseline sharing rate of about 40%, the experiment's statistical power would be rather poor if one assumes the effect to be very small, i.e. our intervention leading to a 5 percentage points increase in code sharing rates (Cohen's *d* = 0.1). In that case, the statistical power for the effort and framing treatment would be 49.1% (*N* = 1,028) and only 30.9% for the appeal treatment (*N* = 514). Assuming a small effect, i.e. our intervention leading to an increase in code sharing rates by 10 percentage points (*d* = 0.2), statistical power is already quite high (94.3% for the binary treatments, 73.8% for the four-level treatment). Finally, for upper medium (*d* = 0.4) and large effect sizes (*d* = 0.8), the statistical power is excellent for all treatments (<99%). Admittedly, these power estimates rely on the assumption of no interaction effects between treatments, i.e. that we tested our main effects in different (independent) samples. If this condition is not met, statistical power would be lower than estimated. However, neither theoretical considerations nor results from the interacted linear probability model reported in Tab. D point towards interactions playing any meaningful role in our study. Taken together, our field experiment's statistical power seems more than adequate (i.e. >80%, [20]) to detect any main effect that would improve code sharing behavior substantially."

*** Minor Comments ***

1. I downloaded and was able to successfully replicate the figures and tables using the same code and data as provided by the authors. The data has a last modified date of 2023-03-30. I then followed up on the pre-registration on "https://osf.io/bqjcz". The registration

was created on 2022-07-06. The only unfortunate thing I can note (and this fact is by no means unique to this project) that it is not possible for the reviewer to verify if the registration was done prior to sample collection with the replication package as is. To this end, I must attach less meaning to the pre-registration than I would be able to otherwise. This can be remedied easily. Line 188 of the manuscript does mention that data collection began on 2022-07-06. I would appreciate some/any method of verification of the emails / replies being sent / coming in after the pre-registration was released.

We greatly appreciate your effort to reproduce our analysis and fully support the request for transparency on the data collection process. To address this concern, we added Fig. A on "Documentation of data collection" to our supplement, where we provide daily information on the number of emails sent/received during our field experiment between July 2022 and February 2023. We believe this is the most convincing way of demonstrating that data collection did, indeed, begin *after* preregistration. Any other piece of evidence either seems easily manipulable (e.g. screenshots of emails), or impractical due to technical reasons (i.e. email logs, which are stored only for 7 days on our university's server, meaning they were no longer accessible at the time of revision).
The revised manuscript points readers to the new figure in the appendix:

> "Following the recommendations by the ethics board, all authors who had responded to any of our emails were debriefed on 17 January 2023 regarding the experimental nature of our request unless they had explicitly objected to receiving any further emails. Fig. A in S1 File provides a breakdown of the daily inflow and outflow of emails between 6 July 2022 and 17 January 2023." (l. 210-214)

2. Quotation marks, particularly opening quotations are often "backwards".

Quotation marks have been corrected throughout the manuscript.

3. Line 13. I believe on line 13 the authors mean "on the authors' side". Typo in line 303 "a long time ago"

Both typos have been corrected.

4. Line 20. The following should be mention/cited here, as it is a well-known and publicized example of this.

Silberzahn, R., & Uhlmann, E. L. (2015). Crowdsourced research: Many hands make tight work. Nature, 526(7572), 189-191.

Silberzahn, R., Uhlmann, E. L., Martin, D. P., Anselmi, P., Aust, F., Awtrey, E., ... & Nosek, B. A. (2018). Many analysts, one data set: Making transparent how variations in

analytic choices affect results. Advances in Methods and Practices in Psychological Science, 1(3), 337-356.

We included both references in the manuscript as well as this more recent many-analysts study:

- Breznau, Nate, Eike Mark Rinke, Alexander Wuttke…. 2022. "Observing Many Researchers Using the Same Data and Hypothesis Reveals a Hidden Universe of Uncertainty." Proceedings of the National Academy of Sciences of the United States of America 119(44):e2203150119. doi: 10.1073/pnas.2203150119.

To acknowledge the lively debate these studies have sparked across the social sciences, we also included the following two references discussing the validity of many-analysts studies:

- Auspurg, Katrin, and Josef Brüderl. 2021. "Has the Credibility of the Social Sciences Been Credibly Destroyed? Reanalyzing the 'Many Analysts, One Data Set' Project." Socius: Sociological Research for a Dynamic World 7:237802312110244. doi: 10.1177/23780231211024421.
- Auspurg, Katrin, and Josef Brüderl. 2023. Is Social Research Really Not Better Than Alchemy? How Many-Analysts Studies Produce "A Hidden Universe of Uncertainty" by Not Following Meta-Analytical Standards. preprint. MetaArXiv. doi: 10.31222/osf.io/uc84k.

The revised section in the manuscript now reads:

> "Given the complexity of analyses, a dataset may yield very different conclusions depending on how it is processed and analyzed. This has been demonstrated by a number of many-analysts studies [9–11] which sparked lively debate across the social sciences [12,13]." (l. 19-22)

Reply to Reviewer #2:

1. (p.2:) You write "In practice, replications hinge on the availability of an original study's code and data." I would argue that this is about reproducibility, and that replication involves new data. I think this is how most psychologists would define replication (including the work of Brian Nosek and others). Some discussion of reproducibility vs replicability might make sense to include.

   We agree that the sentence should read "In practice, reproductions hinge…" and have corrected the wording in the revised manuscript.

   It is undoubtedly true that replications are surrounded by considerable terminological confusion. Although a common terminology would clearly be desirable, we feel it is beyond the scope of our paper to provide the in-depth discussion that would be needed to cover the various definitions and conceptualizations across fields and disciplines. Instead, we point readers to the following helpful paper:

   - Barba, Lorena A. 2018. "Terminologies for Reproducible Research." Working Paper. doi: 10.48550/ARXIV.1802.03311.

   The revised manuscript reads:

   > "Against this backdrop, both conceptual and direct replications - the latter sometimes being called reproductions [7] - have been deemed crucial for determining whether findings are credible and deserve to enter the stock of scientific knowledge [8]." (l. 7-10)

2. (p.2:) When writing about publishing research data being a step towards more open science, I guess this also depends on how "true" we think the data is in terms of whether we only get to see the p-hacked data or not (i.e. outcome variables that were not significant are not included etc).

   We agree that credible science requires full transparency throughout the *entire* research process and that open data alone does not rule out p-hacking, HARKing, and other questionable research practices. Therefore, we strongly encourage current developments in publication practices and formats which attempt to remedy these problems (i.e. preregistrations and registered reports). We refrained from providing a more detailed discussion of this aspect in the manuscript due to space constraints and our focus on code sharing.

3. (p.3, first paragraph in "state of the art":) Here it could be interesting to note that journals increasingly have Data Editors (at least in economics and political science) who go

through the data and code to make sure that the code runs and leads to the same results as in the paper. For example, the journals of the American Economic Association only conditionally accept papers before the data editors have approved the data and code, which timing-wise makes sense.

We revised the manuscript to acknowledge recent developments at the journal level, i.e. the role of data editors. The section includes the following reference:

- Vilhuber, Lars. 2019. "Report by the AEA Data Editor." AEA Papers and Proceedings 109:718–29. doi: 10.1257/pandp.109.718.

The revised manuscript reads:

> "To tackle the unavailability of research material, some journals (i.e. by the American Economic Association) have recently started requiring authors to provide all data and code files upon submission. These replication packages are then checked by a designated data editor to ensure reproducibility [23]." (l. 51-55)

4. Theoretical background: Do you consider all framing nudges? I personally do not really see the need to call the different versions in your experiment nudges, but this is of course up to you. When citing references 49-51 it looks in the main text like those studies were on code sharing PGGs which is not the case since they are on standard ones – perhaps that could be clarified?

We consider our experimental treatments nudges as they meet the definition by Thaler and Sunstein (2008): They constitute minor yet deliberate alterations to the wording of our request (i.e., the individual researcher's "choice architecture") that aim to steer researchers towards predictable behavior (i.e., code sharing). The concepts of framing and nudging overlap to a large degree, but are not fully congruent (see, e.g., Hall 2016 [10.1093/oxfordhb/9780199685271.013.15]). We did not engage in a detailed discussion of these subtle differences for the benefit of conciseness.

We recognize that the positioning of references 49-51 might not have been ideal and appreciate your scrutiny. The refined version of the manuscript now reads:

> "For public good games, evidence suggests that participants are generally more likely to cooperate if a choice situation is framed positively [56–58]. We expect this applies to code sharing, too, and formulate a framing hypothesis." (l. 117-120)

5. In relation to H2.3: Perhaps a bit related to this is the paper by Christensen et al. who find that data sharing is positively related to citations (Christensen G, Dafoe A, Miguel E,

Moore DA, Rose AK (2019) A study of the impact of data sharing on article citations using journal policies as a natural experiment. PLoS ONE, 14(12): e0225883.

We included the suggested reference in the following section:

> "As code sharing has rarely been investigated as a distinct phenomenon, we pertain to survey research on attitudes towards data sharing to identify three potential appeals: compliance with common norms [43,60], altruistic preferences [43,61] and actual career benefits via rightful acknowledgment [43,62–64]." (l. 127-131)

6. (p.7:) I very much appreciate the transparency in how deviations from the pre-analysis plan are listed. From my understanding of reading them they are pretty minor, which was reassuring, but when reading the main text I didn't realize that so then I was a bit more worried (perhaps due to seeing so many papers in the past where there are serious deviations that are not justified or discussed). Perhaps this could be clarified in a footnote in the main text where the deviations are very briefly described?

We are pleased to hear that our attempt to ensure full transparency was well received. As PLOS One does not allow the use of footnotes, we have decided to emphasize the small number and minor nature of all deviations in the main text:

> "All hypotheses and the research design of this study have been preregistered (https://osf.io/bqjcz). Deviations from the preregistration occurred in four very minor instances (e.g. adapting the language of correspondence based on authors' reply). Any deviations are listed, described and justified in Table B in S1 File." (l. 159-162)

7. (p.9:) Given potential power problems, why did you decide to have so many treatments? Some more discussion here would be great.

In line with your suggestion, we added a section on statistical power. We would like to point out, though, that the statistical power for estimating our main effects is not affected by the number of *treatments* but rather by the number of *levels per treatment* (which we set deliberately low when choosing a 2 x 2 x 4 design). The power for estimating the effort effect, for instance, remains stable no matter if we split the high-/low-effort group further into subgroups based on other treatments (assuming there are no interaction effects).

The revised manuscript includes the following sentence:

> "Assuming a moderately large effect, i.e. our treatment leading to an increase in code sharing rates by 15 percentage points (d = 0.3), the statistical power for both two-level and four-level treatments is more than adequate (96.25% and 99.93%, respectively; see Text 1 in S1 File for a detailed discussion on statistical power)." (l. 252-256)

The respective section in the Supplemental Material reads:

> "Given our sample size and the observed baseline sharing rate of about 40%, the experiment's statistical power would be rather poor if one assumes the effect to be very small, i.e. our intervention leading to a 5 percentage points increase in code sharing rates (Cohen's d = 0.1). In that case, the statistical power for the effort and framing treatment would be 49.1% (N = 1,028) and only 30.9% for the appeal treatment (N = 514). Assuming a small effect, i.e. our intervention leading to an increase in code sharing rates by 10 percentage points (d = 0.2), statistical power is already quite high (94.3% for the binary treatments, 73.8% for the four-level treatment). Finally, for upper medium (d = 0.4) and large effect sizes (d = 0.8), the statistical power is excellent for all treatments (<99%). Admittedly, these power estimates rely on the assumption of no interaction effects between treatments, i.e. that we tested our main effects in different (independent) samples. If this condition is not met, statistical power would be lower than our estimates. However, neither theoretical considerations nor results from the interacted linear probability model reported in Tab. D point towards interactions playing any meaningful role in our study. Taken together, our field experiment seems more than adequately equipped (i.e. >80%, [20]) to detect any main effect that would improve code sharing behavior substantially."

8. (p.11, line 262:) What is the size of this share?

   The revised manuscript includes the number of researchers who needed clarification what was meant by research code:

   > "If authors indicated confusion about what we meant by research code (n = 22), we sent a follow-up email providing examples and clarification." (l. 279-281)

9. (p.13, line 319:) I maybe misunderstand something, but a z-value of 2.287 should have a low p-value – is there an error here? (and maybe elsewhere? I haven't checked the other ones.) The null hypothesis is no effect so even though the result is in the wrong direction, it is an effect that is statistically significant.

   Many thanks for pointing us towards this potential source of confusion. Based on

previous research, we expected that positive framing would *increase* researchers compliance with our request (directional hypothesis). To test this hypothesis adequately, we had to employ a left-tailed test. Given that observed compliance was, in fact, substantially *lower* among those who received a positive framing ($\hat{p}_{\text{positive}} = 0.340$ vs. $\hat{p}_{\text{negative}} = 0.409$), the test does correctly yield $z = 2.287$ and $p = 0.989$. We understand that this might seem rather confusing at first sight. Had we been interested in the mere *existence* of an effect (as opposed to its *direction*), the appropriate two-tailed test would, indeed, yield a significant p-value of 0.0222.

Our results may be reproduced using the do-file "03_appendix_tab_prtest_mainresults".
